# Biomechanical effects of traditional Chinese exercises on scoliosis: a combined finite element and surface electromyography analysis

**DOI:** 10.3389/fbioe.2026.1895714

**Published:** 2026-10-09

**Authors:** Boyu Zhang, Tan Liu, Haojie Wang, Dong Liu, Pengren Luo, Mengxiang Li, Jiaji Zhang, Jianguo Li, Minghui Zhuang, Chong Lu, Tao Han, Zhefeng Jin, Liguo Zhu

**Affiliations:** 1 The Third Department of Sports Medicine, Wangjing Hospital, China Academy of Chinese Medical Sciences, Beijing, China; 2 China Institute for History of Medicine and Medical Literature, China Academy of Chinese Medical Sciences, Beijing, China; 3 Department of Tuina, Shanxi Provincial Integrated TCM and WM Hospital, Taiyuan, Shanxi, China; 4 Beijing Key Laboratory of Orthopedics of Traditional Chinese Medicine, Beijing, China

**Keywords:** idiopathic scoliosis, muscle activation symmetry, spinal biomechanics, surface electromyography, traditional Chinese exercises

## Abstract

**Objective:**

To investigate the effects of two specific traditional Chinese exercise movements—“Lu Di” and “Jiu Gui Ba Ma Dao”—on spinal biomechanics and trunk muscle sEMG characteristics, elucidating their potential corrective mechanisms for idiopathic scoliosis.

**Methods:**

A Lenke type 1 scoliosis finite element model (T1-S1) was developed by applying mesh deformation techniques to a healthy adult spine model. Kinematic parameters of the spinous processes were acquired from 10 healthy subjects performing the two exercises using inertial sensors and applied as boundary conditions for the FEA. Vertebral and intervertebral disc stress distributions were analyzed under a fixed loading angle during the “Lu Di” movement. Concurrently, sEMG signals from the bilateral erector spinae and multifidus were recorded in patients with Lenke type 1 scoliosis during the exercises to evaluate muscle activation timing and inter-limb symmetry.

**Results:**

FEA demonstrated that complex movement loading induced favorable adjustments in stress distribution and deformation patterns, notably reducing disc stress on the concave side of the apical vertebra. sEMG analysis revealed that both exercises modulated trunk muscle activation levels and bilateral symmetry. They effectively increased force output in the convex musculature without inducing significant fatigue, while appropriately enhancing activation on the concave side.

## 1 Introduction

Adolescent idiopathic scoliosis (AIS) is a complex three-dimensional spinal deformity characterized by coronal lateral curvature, altered sagittal profile, and axial vertebral rotation (Labrom et al., 2021). Affecting a substantial adolescent population globally, AIS causes cosmetic concerns, back pain, cardiopulmonary compromise, and reduced quality of life, imposing significant burdens on patients and their families (Li et al., 2024; Torén and Diarbakerli, 2022). Despite decades of research, the exact etiology, progression patterns, and core corrective mechanisms of conservative treatments for AIS remain incompletely elucidated (Cheng et al., 2015).

In exploring the biomechanical mechanisms of scoliosis, finite element (FE) analysis has emerged as an indispensable tool. It accurately simulates complex spinal geometry and material properties to quantify the internal stress, strain, and deformation of vertebrae, intervertebral discs, and ligaments under various loads (Wang et al., 2023). Historically, FE studies have predominantly focused on the biomechanics of passive correction strategies, such as surgical internal fixation, bracing, and traction therapies (Shi et al., 2025; Zhang et al., 2025a; Yang et al., 2025). While these studies have significantly advanced our understanding of spinal mechanical responses and stress distributions under static or external loads, they largely overlook the vital role of active neuromuscular control in modulating internal loads. In contrast, movement-based rehabilitation relies heavily on internal loading and synergistic muscle activation induced by active patient motion, which form the true biomechanical and physiological basis of its efficacy. Therefore, the underlying mechanisms of active exercise therapies warrant further in-depth investigation (Adams and Dolan, 2005).

Exercise-based interventions constitute a well-established pillar of conservative management for adolescent idiopathic scoliosis (AIS). Among these, physiotherapeutic scoliosis-specific exercises (PSSE)—notably the Schroth method—are widely endorsed as first-line non-surgical interventions to enhance postural control, restore muscular symmetry, and reinforce spinal stability (Liu et al., 2025; Jia et al., 2025). Systematic reviews support their efficacy: PSSE, whether delivered alone or in combination with bracing, demonstrates clinically meaningful effects in slowing curve progression and improving health-related quality of life—particularly in patients with mild-to-moderate AIS (Cobb angle 10°–45°) (Karımi et al., 2021). Furthermore, emerging evidence suggests that habitual physical activity—not limited to scoliosis-specific regimens—may influence sagittal spinal alignment, with longitudinal studies reporting associations between regular training and reduced thoracic kyphosis or increased lumbar lordosis; such findings imply that cumulative mechanical loading contributes to adaptive postural remodeling (Bingöl et al., 2025).

Despite this progress, clinical evaluation remains predominantly reliant on macroscopic geometric parameters—primarily Cobb angle, apical vertebral translation, and axial rotation—while quantitative assessments of tissue-level biomechanics (e.g., intervertebral disc stress distribution, facet joint loading) and rigorous characterization of neuromuscular control strategies (e.g., muscle activation sequencing, proprioceptive integration) remain scarce. Many conventional PSSE protocols necessitate specialized equipment or therapist-guided manual corrections, introducing confounding spinal loads from both body weight and externally applied forces—factors that complicate parametric modeling and limit translational fidelity in computational simulations. Traditional Chinese Exercises (TCEs)—such as Baduanjin and Wuqinxi—represent a distinct movement paradigm: low-cost, self-administered practices requiring no equipment, characterized by intentional mental focus, diaphragmatic breathing, and multiplanar, spiraling spinal articulation (Li P. et al., 2025). These modalities prioritize integrated spinal mobility and dynamic postural regulation across functional tasks; however, their biomechanical impact in AIS populations has thus far been reported almost exclusively through qualitative observation or subjective appraisal, with minimal objective quantification of kinematic, kinetic, or neuromuscular outcomes (Chen et al., 2023).

This study developed an integrated framework combining inertial motion capture, surface electromyography (sEMG), and finite element (FE) modeling to evaluate two traditional corrective exercises—“Lu Di” and “Jiu Gui Ba Ma Dao”—in adolescent idiopathic scoliosis (AIS). Three-dimensional angular data from inertial measurement units (IMUs) were applied as displacement-driven boundary conditions to a geometrically idealized Lenke 1 scoliosis model, enabling posture-specific biomechanical simulation of “Lu Di.” sEMG was used to quantify paraspinal muscle activation and side-to-side recruitment patterns during both exercises (Suo et al., 2024).

Preliminary results showed distinct kinematic and neuromuscular profiles between the two movements. For “Lu Di,” FE analysis indicated a redistribution of apical disc stress toward a more balanced pattern, whereas interpretations for “Jiu Gui Ba Ma Dao” were limited to kinematic and EMG observations. sEMG differences between the concave and convex sides did not persist after correction for multiple comparisons and were therefore considered exploratory. By coupling subject-specific motion and muscle activation data with computational modeling, this work provides preliminary biomechanical evidence that active postural exercises can modulate spinal loading and paraspinal recruitment. Future longitudinal studies are required to determine whether these acute responses translate into structural or functional benefits in conservative AIS management.

## 2 Methods

### 2.1 Development of the AIS finite element model

Geometric data for the baseline finite element (FE) model were acquired from a healthy adult female (height: 166 cm) with no history of scoliosis or congenital spinal disorders. Three-dimensional geometries of the T1-S1 vertebrae and intervertebral discs (differentiating the annulus fibrosus and nucleus pulposus) were reconstructed using Mimics software. Major spinal ligaments were modeled as tension-only spring elements. The material properties (elastic modulus and Poisson’s ratio) for cortical bone, cancellous bone, endplates, annulus fibrosus, and nucleus pulposus are detailed in Table 1. The model used first-order tetrahedral elements (9,643,589 elements, 1,928,230 nodes; global mesh size 2 mm, with local refinement at endplates and facets). A mesh convergence study showed <3% change in peak von Mises stress and <2% change in segmental rotation between the final two mesh densities (Zhang et al., 2023). The sacrum was fully fixed at the inferior endplate of S1 in all six degrees of freedom to represent rigid pelvic support. Gravitational loading was not included in the current quasi-static analysis and is acknowledged as a limitation. No compressive follower load was applied; loading was limited to a 7.5 Nm pure moment combined with prescribed displacements. The rib cage was not modeled in detail; the ribs and sternum were omitted to reduce model complexity, and rotational stiffness was therefore governed solely by the intervertebral discs, facet joints, and posterior elements. A surface-to-surface frictional contact was defined between opposing facet articular surfaces, with a friction coefficient of 0.46, consistent with experimental data on cartilage sliding behavior. The baseline model was validated under pure moments (flexion/extension, lateral bending, and axial rotation) by comparing the range of motion (ROM) against published *in vitro* and FE data. Subsequently, the validated healthy spine model was geometrically deformed using SolidWorks to construct a Lenke type 1 AIS model (predominantly thoracic curve). The deformity was characterized by a main thoracic Cobb angle of approximately 20°, consistent with moderate AIS. The apical vertebra was rotated approximately 5° according to the Raimondi method. Global sagittal balance was preserved, with thoracic kyphosis increased to 40° and lumbar lordosis set at 39°. Geometric changes included reduced disc height on the concavity, mild wedging of the apical vertebral body, and laterally shifted spinous processes. These parameters were implemented using landmark-based surface warping constrained by typical scoliotic morphology. This study was approved by the Ethics Committee of Wangjing Hospital, China Academy of Chinese Medical Sciences (Approval No. WJEC-KT-2025-065-P001).

**TABLE 1:** Material properties assigned to the spinal finite element model.

Component	Young’s modulus (E, MPa)	Poisson’s ratio (ν)
Cortical bone	12,000	0.3
Cancellous bone	100	0.3
Endplate	24	0.4
Articular cartilage	25	0.25
Nucleus pulposus	1	0.49
Annulus fibrosus	4	0.3

### 2.2 Kinematics and surface electromyography acquisition

Participants: Ten healthy adults proficient in traditional Chinese exercises (TCEs) (Cobb angle <10°) and 10 patients with Lenke type 1 AIS (thoracic Cobb angle ≥10°) were recruited. Individuals with other spinal pathologies or joint hypermobility were excluded. Participant demographics and curve characteristics are summarized in Table 2. All enrolled participants completed the experimental session; no datasets were excluded from analysis.

**TABLE 2:** Basic information of participants.

Variable	Healthy controls	AIS patients
Height (m)	1.67 ± 0.02	1.68 ± 0.04
Weight (kg)	58.70 ± 3.18	51.78 ± 6.5
BMI (kg/m^2^)	20.66 ± 0.66	18.18 ± 1.65
Thoracic Cobb angle (°)	—	14.20 ± 3.45

Kinematic Tracking: Kinematic data were recorded using inertial measurement units (IMUs) attached over the spinous processes of T1, T6, T12, L3, and L5, with an additional sensor on the sacrum serving as a reference point. The extreme spinal positions during static standing and each decomposed phase of the “Lu Di” and “Jiu Gui Ba Ma Dao” exercises were continuously tracked. Additionally, an OptiTrack motion capture system (Prime 13 cameras, 1.3 megapixels, 0.01 mm precision) was employed to record the movements for virtual model demonstrations.

Surface Electromyography (sEMG): Muscle activity was recorded using a wireless sEMG system (BTS Bioengineering, Italy) at a sampling frequency of 1,000 Hz. Surface electrodes were placed bilaterally over the erector spinae and multifidus muscles at the level of the apical vertebra in each patient with AIS. Before electrode placement, the skin was lightly abraded and cleaned with alcohol in accordance with SENIAM recommendations to reduce skin impedance and improve signal quality.

Raw sEMG signals were band-pass filtered between 20 and 450 Hz, and a 50-Hz notch filter was applied to suppress power-line interference. No full-wave rectification was performed. Root mean square (RMS) amplitude was calculated directly from the filtered sEMG signals using a 200-ms sliding window. Median frequency (MF) was derived from the power spectrum of the filtered, unrectified signals using fast Fourier transform (FFT) over consecutive 1-s epochs. To reduce inter-individual variability, RMS and MF values obtained during the exercises were normalized to the corresponding baseline values recorded during static standing. Signal quality was visually inspected, and segments contaminated by obvious motion artifacts or baseline drift were excluded from subsequent analysis.

During each traditional Chinese exercise (TCE), participants were instructed to maintain the terminal position for 3 s, and the normalized RMS and MF values during these terminal phases were extracted for bilateral comparison between the concave and convex sides. To standardize the corrective movement direction, each participant performed the exercise in a direction determined according to the individual scoliotic curve pattern. For example, a participant with a right thoracic convexity performed the prescribed lateral-bending movement toward the right.

### 2.3 Boundary and loading conditions for FE analysis

FE simulations were driven by a combination of kinematic displacements and mechanical loads. The *in vivo* 3D segmental angles relative to the pelvis, captured via IMUs, were applied to the vertebrae as displacement boundary conditions to replicate specific postures. This represents an externally applied postural correction, not a muscle-driven dynamic motion. Concurrently, a pure moment of 7.5 Nm (consistent with previous literature) was applied to the T1 superior endplate in the direction corresponding to each decomposed movement phase. Because actual traditional Chinese exercises (TCEs) involve coupled multidirectional movements (e.g., simultaneous flexion, lateral bending, and rotation), transient dynamic simulations were omitted. Instead, static analyses were conducted at the maximal extreme positions. Thus, the results describe the passive mechanical environment under corrective positioning, complementing rather than replacing musculoskeletal models with muscle–tendon forces. Three primary scenarios were simulated: (1) static standing, (2) the AIS model at the “Lu Di” extreme position, and (3) the AIS model at the extreme position of the specified spinal movement.

### 2.4 Outcome measures and data analysis

Finite Element Metrics: FEA was conducted on “Lu Di” using Abaqus 6.14. We evaluated the overall von Mises stress distribution across the intervertebral discs, with a specific focus on the disc immediately inferior to the apical vertebra. To quantify stress asymmetry, disc elements were partitioned into left and right regions along the sagittal plane using nodal coordinates. The asymmetry ratio of the mean stress between the left and right sides of the discs (specifically those adjacent to the end vertebrae) was calculated under different loading conditions.

Kinematic and sEMG Processing: IMU angular data from healthy subjects were denoised, fitted, and averaged to derive representative integer values across the three anatomical planes. For sEMG analysis, time-domain (Root Mean Square [RMS]) and frequency-domain (Median Frequency [MF], Mean Power Frequency [MPF]) parameters were extracted. To account for individual differences, all dynamic sEMG metrics during the exercises were normalized against the baseline values obtained during static standing.

Data analysis and visualization were performed using SPSS 26.0 and GraphPad Prism 5.0. Descriptive statistics for the sEMG variables are presented as mean ± standard deviation (SD). Because concave- and convex-side measurements were obtained bilaterally from the same participants, all inter-side comparisons were treated as paired observations. The normality of the paired differences (concave side minus convex side) was assessed using the Shapiro–Wilk test. When the paired differences were normally distributed, concave-versus convex-side comparisons were performed using paired-samples t tests; otherwise, the Wilcoxon signed-rank test was used. To account for multiple concave-versus-convex comparisons, P values within each exercise were adjusted using the Holm procedure to control the family-wise error rate. Both unadjusted and Holm-adjusted P values are reported. Effect sizes were calculated as Hedges’ g with 95% confidence intervals (CIs). All tests were two-sided, and a Holm-adjusted P < 0.05 was considered statistically significant.

## 3 Results

### 3.1 Demographic and clinical characteristics of participants

The study cohort comprised 10 healthy controls (5 males and 5 females) and 10 patients with Lenke type 1 AIS (2 males and 8 females). There were no significant differences in age, height, or weight between the two groups. In the AIS group, the mean thoracic Cobb angle was 14.20° ± 3.45° confirming a diagnosis of Lenke type 1 scoliosis. Detailed demographic and clinical profiles of all participants are summarized in Table 2.

### 3.2 Kinematic results from sensor measurements

The 3D angular displacements during the two traditional Chinese exercises (TCEs) were averaged across the 10 subjects for both left- and right-sided movements. Each exercise was decomposed into primary directional components: flexion/extension, lateral bending, and axial rotation. The mean angular values at the maximal positions for each direction are summarized in Table 3.

**TABLE 3:** Kinematic characteristics and vertebral angles at extreme positions of the two traditional Chinese exercises.

Movement direction	Spinal segment	Jiu Gui Ba ma Dao (°)	Lu di (°)
Flexion	T1	88	50
	T6	84	45
	T12	74	40
	L5	35	30
Lateral bending (Left/Right)	T1	20	28
	T6	19	25
	T12	18	20
	L5	15	16
Axial rotation (Left/Right)	T1	85	60
	T6	80	40
	T12	58	30
	L5	48	24
Primary motion planes		Axial and sagittal planes	Axial, sagittal, and coronal planes
Dominant segments for axial rotation		Upper-to-middle thoracic (T1, T6)​	Upper thoracic (T1)
Dominant segments for flexion		Progressively decreasing from superior to inferior	Lower lumbar (L5)

Biomechanical analysis revealed that the “Jiu Gui Ba Ma Dao” movement sequence involves an initial spinal flexion followed by coupled rotation and lateral bending. Conversely, “Lu Di” exhibits a spiral torsion pattern characterized by alternating dominance of the thoracic and lumbar segments, which can be decomposed into spinal flexion followed by concurrent lateral bending and rotation. Changes in acceleration and angular velocity recorded by the IMUs throughout the exercises, along with the synchronized animation models captured via the OptiTrack system, are provided in the Supplementary Materials.

### 3.3 Surface electromyography (sEMG) results

Following normalization to the static standing baseline, the “Lu Di” exercise showed consistent bilateral differences in multifidus RMS activation. At the terminal ranges of flexion (1.71 ± 0.49 vs. 1.55 ± 0.51; unadjusted P = 0.021, Holm-adjusted P = 0.210), lateral bending (2.07 ± 0.91 vs. 1.88 ± 0.83; unadjusted P = 0.011, Holm-adjusted P = 0.121), and axial rotation (2.19 ± 0.87 vs. 1.96 ± 0.76; unadjusted P = 0.006, Holm-adjusted P = 0.072), multifidus RMS was higher on the convex side. Although these differences were statistically significant before multiplicity adjustment, none remained significant after Holm correction. No significant inter-side differences in erector spinae RMS were observed across the three movement directions after correction.

For “Jiu Gui Ba Ma Dao,” no significant inter-side differences in RMS were observed after Holm correction. During lateral bending, the concave-side erector spinae showed higher RMS activation than the convex side (1.57 ± 0.51 vs. 1.45 ± 0.44; unadjusted P = 0.048, Holm-adjusted P = 0.576), but this difference did not remain statistically significant after multiplicity adjustment. No significant inter-side differences in median frequency (MF) were detected for either the erector spinae or multifidus across the examined movement directions during either exercise after Holm correction. The sEMG results are summarized in Tables 4, 5.

**TABLE 4:** Comparison of normalized sEMG metrics at the extreme positions during the “Lu Di” exercise.

sEMGMetric	Direction	Muscle	Concave side	Convex side	Shapiro-Wilk P	t/Z (df)	P-value	Hedges’ g (95%CI)	Holm-adjusted P
RMS	Flexion	ErectorSpinae	1.44 ± 0.32	1.82 ± 0.51	0.278	t = −2.077 (9)	0.068	(−1.274, 0.044)	0.612
		Multifidus	1.55 ± 0.51	1.71 ± 0.49	0.498	t = −2.804 (9)	**0.021**	(−1.541, −0.125)	0.21
	LateralBending	ErectorSpinae	1.86 ± 0.39	2.06 ± 0.42	0.278	t = −1.782 (9)	0.109	(−1.169, 0.115)	0.872
		Multifidus	1.88 ± 0.83	2.07 ± 0.91	0.498	t = −3.201 (9)	**0.011**	(−1.692, −0.213)	0.121
	AxialRotation	ErectorSpinae	2.00 ± 0.41	2.13 ± 0.47	0.854	t = −1.068 (9)	0.313	(−0.927, 0.296)	1
		Multifidus	1.96 ± 0.76	2.19 ± 0.87	0.108	t = −3.596 (9)	**0.006**	(−1.845, −0.299)	−0.072
MF	Flexion	ErectorSpinae	1.11 ± 0.26	1.04 ± 0.17	0.531	t = 1.411 (9)	0.192	(−0.209, 1.041)	0.96
		Multifidus	1.11 ± 0.23	1.10 ± 0.27	0.263	t = 0.494 (9)	0.633	(−0.452, 0.743)	1
	LateralBending	ErectorSpinae	1.32 ± 0.22	1.23 ± 0.22	0.531	t = 1.576 (9)	0.149	(−0.166, 1.098)	0.894
		Multifidus	1.34 ± 0.37	1.33 ± 0.42	0.263	t = 0.283 (9)	0.783	(−0.511, 0.678)	1
	AxialRotation	ErectorSpinae	1.30 ± 0.22	1.26 ± 0.30	0.515	t = 0.653 (9)	0.541	(−0.413, 0.787)	1
		Multifidus	1.41 ± 0.44	1.35 ± 0.38	0.036	Z = −1.580 (/)	0.114	(−0.268, 0.963)	0.872

Bold value represents p < 0.05.

**TABLE 5:** Comparison of normalized sEMG metrics at the extreme positions during the “Jiu Gui Ba Ma Dao” exercise.

sEMGMetric	Direction	Muscle	Concave side	Convex side	Shapiro-Wilk P	t/Z (df)	P-value	Hedges’ g (95%CI)	Holm-adjusted P
RMS	Flexion	ErectorSpinae	1.32 ± 0.57	1.22 ± 0.42	1.000	t = 1.376 (9)	0.202	(−0.217, 1.029)	1
		Multifidus	1.26 ± 1.86	1.31 ± 2.22	0.126	t = −1.623 (9)	0.139	(−1.114, 0.154)	1
	LateralBending	ErectorSpinae	1.57 ± 0.51	1.45 ± 0.44	0.875	t = 2.281 (9)	**0.048**	(0.005, 1.348)	0.576
		Multifidus	2.01 ± 0.58	2.13 ± 0.69	0.383	t = −1.296 (9)	0.227	(−1.002, 0.237)	1
	AxialRotation	ErectorSpinae	1.78 ± 0.58	1.67 ± 0.49	0.908	t = 2.182 (9)	0.057	(−0.217, 1.029)	0.627
		Multifidus	2.10 ± 0.60	2.26 ± 0.76	0.695	t = −1.548 (9)	0.156	(−1.114, 0.154)	1
MF	Flexion	ErectorSpinae	1.20 ± 0.30	1.15 ± 0.30	0.324	t = 0.758 (9)	0.468	(−0.379, 0.826)	1
		Multifidus	1.83 ± 0.50	1.87 ± 0.49	0.23	t = −0.475 (9)	0.646	(−0.737, 0.457)	1
	LateralBending	ErectorSpinae	1.31 ± 0.31	1.23 ± 0.29	0.512	t = 1.301 (9)	0.226	(−0.236, 1.004)	1
		Multifidus	1.95 ± 0.44	2.02 ± 0.45	0.401	t = −0.990 (9)	0.348	(−0.901, 0.317)	1
	AxialRotation	ErectorSpinae	1.43 ± 0.23	1.34 ± 0.23	0.681	t = 1.567 (9)	0.152	(−0.379, 0.826)	1
		Multifidus	2.05 ± 0.48	2.08 ± 0.45	0.798	t = −0.373 (9)	0.717	(−0.737, 0.457)	1

Bold value represents p < 0.05.

### 3.4 Finite element model validation and specifications

The material properties assigned to the various components of the finite element (FE) model are summarized in Table 1. The finite element modeling process is illustrated in Figures 1, 2. Under a pure moment of 7.5 Nm, the calculated spinal range of motion (ROM) across different planes was validated against experimental data, as illustrated in Figures 3, 4 and detailed in Table 6.

**FIGURE 1:**
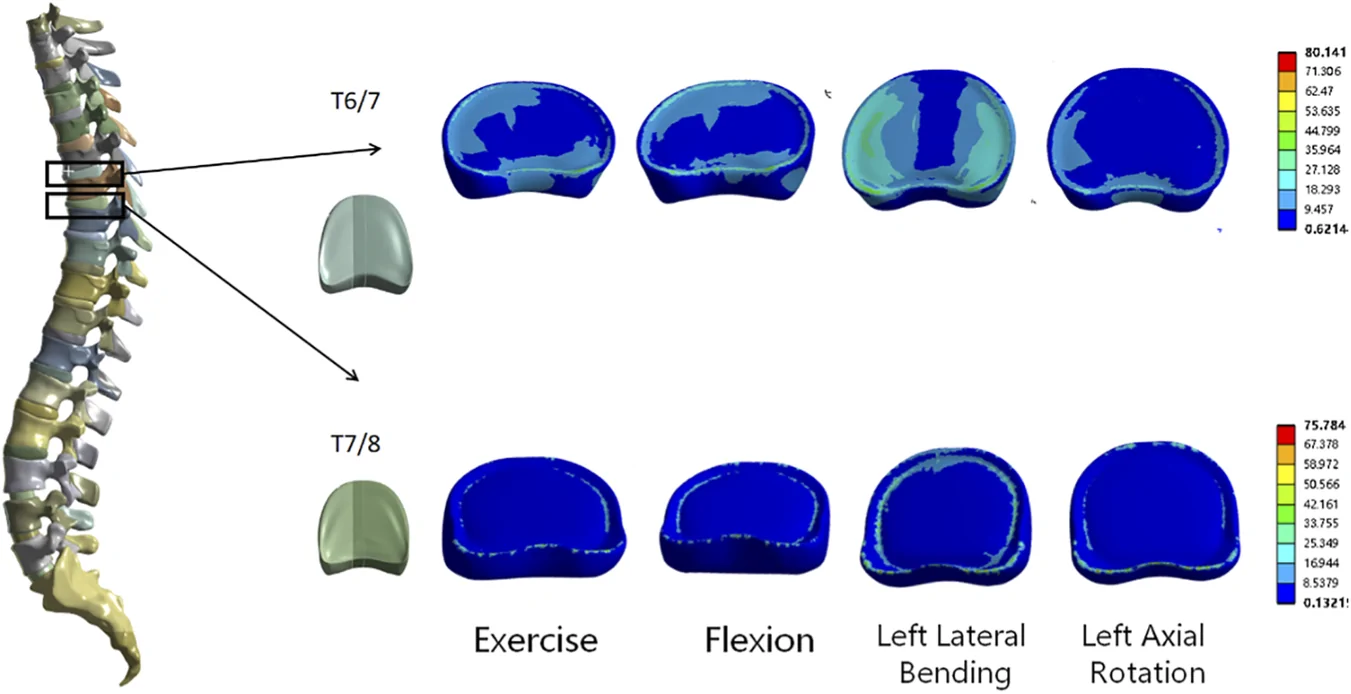
Equivalent (von Mises) stress contour maps of the intervertebral discs adjacent to the apical vertebra during different simulated movements.

**FIGURE 2:**
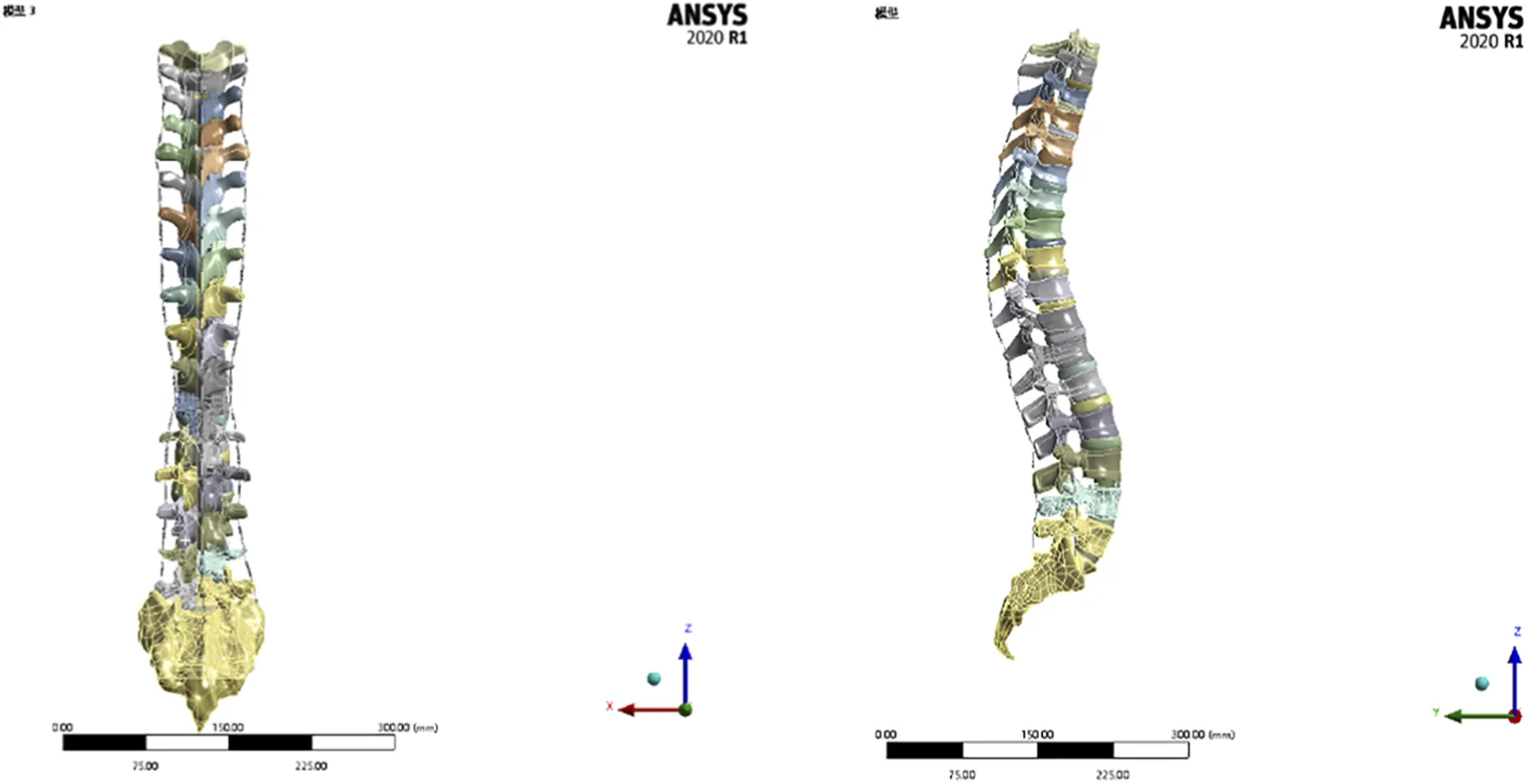
Three-dimensional finite element model of the normal (intact) lumbar spine.

**FIGURE 3:**
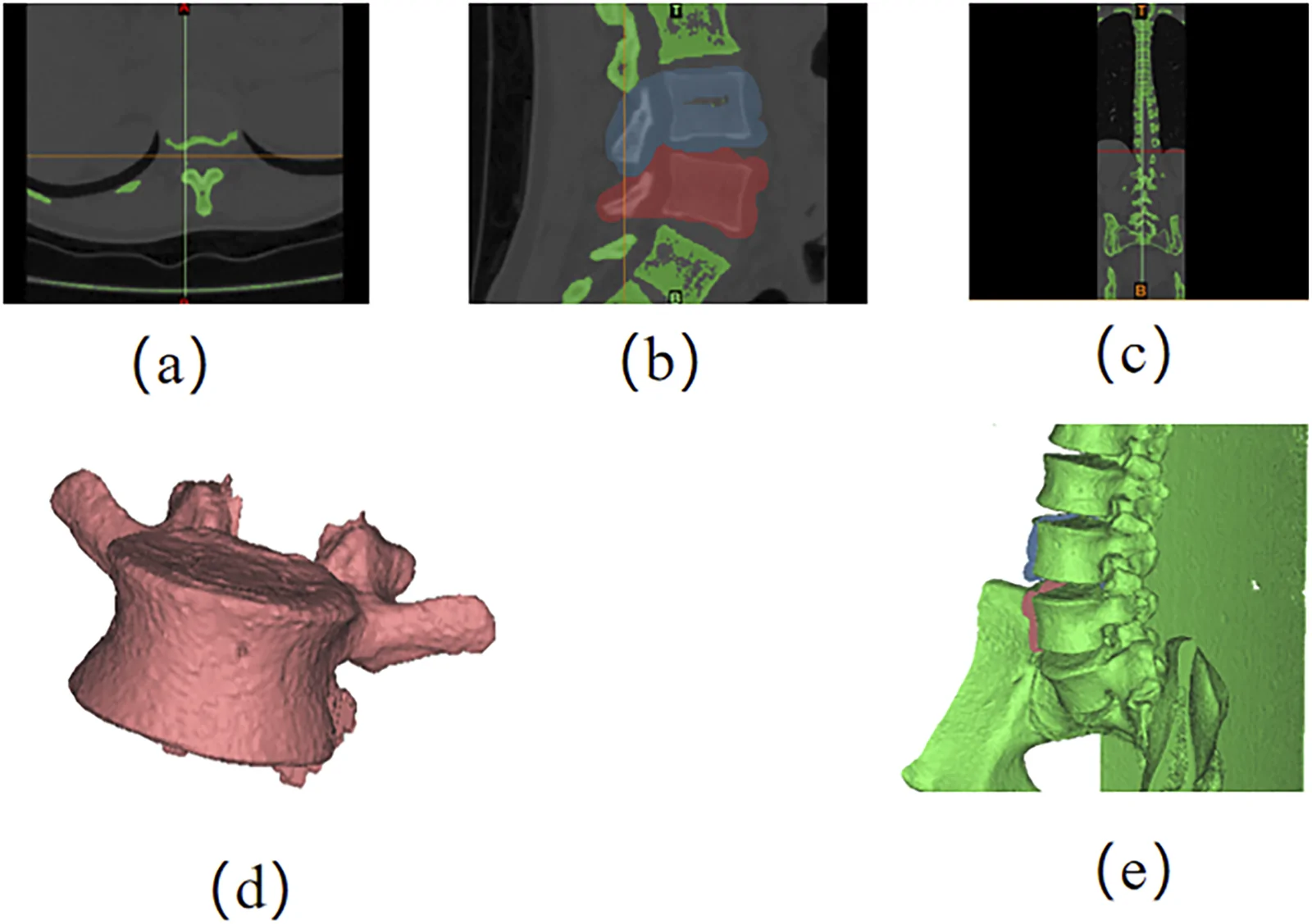
Workflow of the L1–S1 spinal finite element model construction. **(a–c)** Segmentation of anatomical regions of interest (ROI); **(d–e)** Manual mask editing and refinement within Mimics software.

**FIGURE 4:**
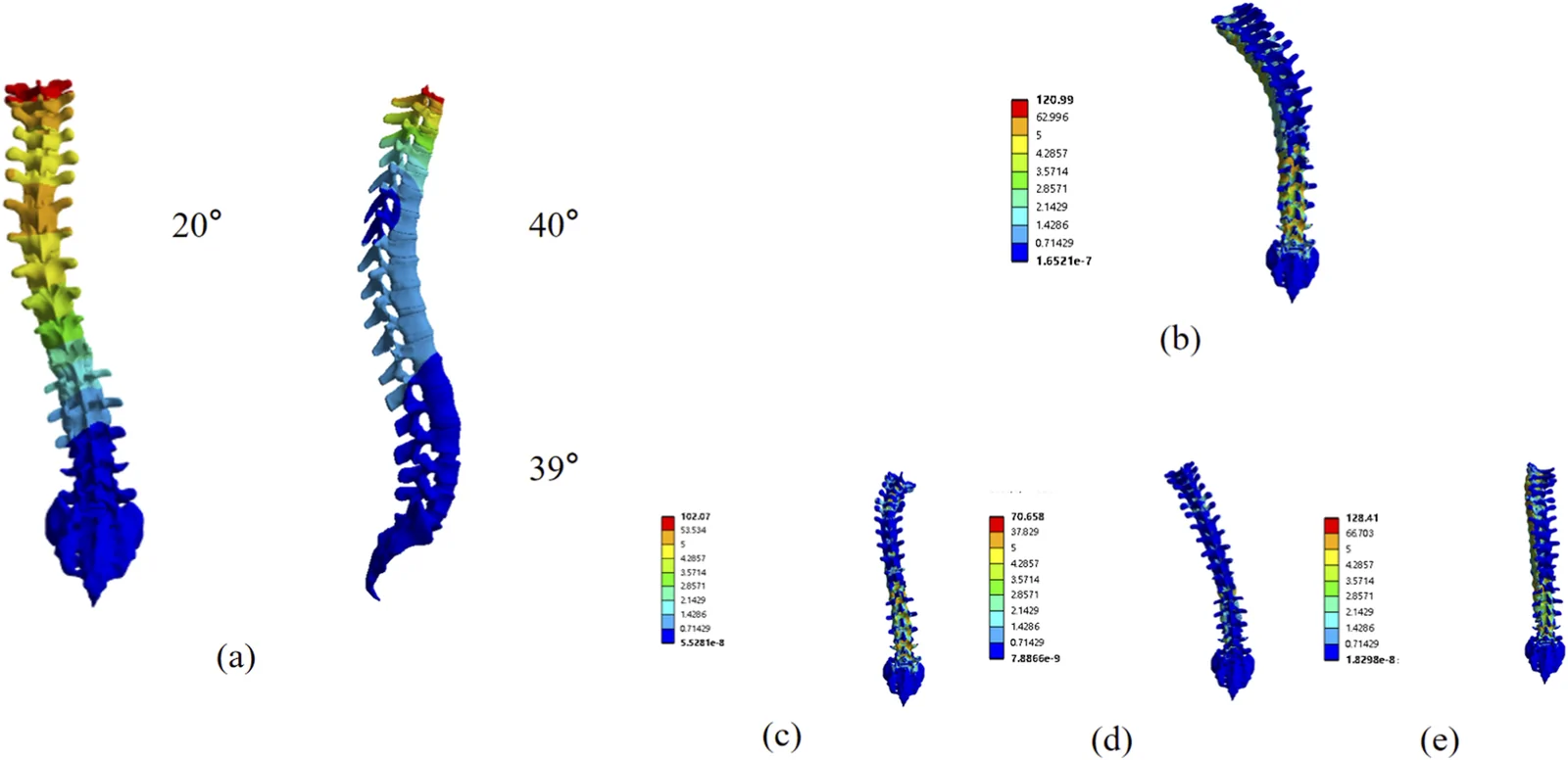
Global deformation and stress contour maps of the scoliosis finite element model. **(a)** Initial state of the scoliosis model with the apical vertebra located at T7; **(b–c)** Finite element views of the spine at the terminal ranges of the prescribed exercises; **(d–f)** von Mises stress distributions (MPa) of the scoliosis model under flexion, left lateral bending, and left axial rotation, respectively.

**TABLE 6:** Predicted ranges of motion (ROM) of the spinal finite element model under 7.5 Nm moment.

Range of motion (ROM, °)					
Loading Direction​	T1-S1	T1-T6	T6-T12	T6-S1	T12-S1
Flexion-extension	103.5	65.9	28.2	37.6	9.4
Lateral bending	109.5	65.9	31.5	43.5	12.0
Axial rotation	124.1	69.5	40.9	54.6	13.7

Regarding the interface definitions within the assembly, tie constraints were utilized to simulate the physiological attachment between the endplates and the vertebral bodies. For the facet joints, a frictional contact interaction was defined between the articular cartilage and the corresponding articular surfaces of the pedicles, with a friction coefficient prescribed at 0.46 to replicate realistic joint sliding. Under compound corrective loading, the peak von Mises stress was 120.99 MPa, localized at the apical vertebra of the thoracic scoliotic segment. Under uni-directional loading, the maxima occurred at distinct spinal levels and followed a consistent left-sided directional pattern: Flexion: 102.07 MPa at the L5 vertebral body; Lateral bending (left): 70.66 MPa at the apical thoracic vertebra; Axial rotation (left): 128.41 MPa at the mid-upper thoracic region (T2 level).

At the terminal position of the “Lu Di” exercise, the T6/7 and T7/8 intervertebral discs exhibited significantly higher stress on the convex (left) side compared to the concave (right) side, indicating the formation of a “stress-reversal” state in the apical region. Under a pure flexion moment, stress remained higher on the concave side (a static characteristic), suggesting that flexion may balance global stress by loading the concavity. Conversely, left lateral bending and left axial rotation amplified the high-stress state on the convex side, with left rotation inducing the greatest bilateral asymmetry (22.86% at the T7/8 level). This demonstrates that both uni-directional movements increase convex-side compression moments. Notably, while the “Lu Di” exercise maintained the therapeutic convex-dominant stress pattern (left > right), its absolute mean stresses on both sides were lower than those observed under pure lateral bending and rotation. This finding suggests that while effectively producing convex-side loading, the “Lu Di” maneuver imposes a lower overall compressive burden on the intervertebral discs compared to single-direction motions. The detailed results are shown in Table 7.

**TABLE 7:** Comparison of mean equivalent (von Mises) stress between left and right sides of T6/7 and T7/8 intervertebral discs at exercise terminal ranges under different loading conditions.

Loading condition	Disc level	Mean left-side stress (MPa)	Mean right-side stress (MPa)	Absolute difference (MPa)	Bilateral asymmetry ratio (%)
Exercise terminal moment	T6/7	3.83	3.22	0.61	15.88%
	T7/8	3.27	2.74	0.53	16.21%
Flexion moment	T6/7	3.54	3.85	−0.31	−8.76%
	T7/8	2.85	3.41	−0.56	−19.65%
Left lateral bending moment	T6/7	4.16	3.37	0.79	18.99%
	T7/8	3.55	2.78	0.77	21.69%
Left axial rotation moment	T6/7	4.64	3.82	0.82	17.67%
	T7/8	3.98	3.07	0.91	22.86%

Values represent deterministic outputs from a single finite element model, not inferential statistical estimates.

## 4 Discussion

### 4.1 Interpretation of main findings

By integrating IMU kinematics, sEMG, and FEA, this study systematically characterized the biomechanical and neuromuscular patterns of two traditional maneuvers, “Jiu Gui Ba Ma Dao” and “Lu Di,” for AIS. Our findings indicate that their distinct movement patterns are the fundamental drivers of differentiated biomechanical responses.

The Lenke 1 AIS model was generated by geometrically deforming a healthy adult template and therefore represents an idealized, rather than patient-specific, reconstruction. Global range of motion under ±7.5 Nm moments aligned with previously reported experimental data (Rohlmann et al., 2001), supporting the model’s kinematic plausibility under the applied loading. This validation confirms overall flexibility and load transfer, but does not imply full biofidelity of anatomical detail or intervertebral stress distribution.

“Jiu Gui Ba Ma Dao” was characterized by thoracic-dominant coordinated rotation. Kinematic analysis showed greater rotational amplitudes in the upper thoracic segments (T1/T6: 85°/80°) than in the lumbar region (48°), indicating that the movement primarily emphasized thoracic rotation rather than lumbar compensation. This thoracic-dominant movement pattern may be relevant to active de-rotation training in AIS patients with vertebral rotation. The sEMG findings further suggested movement-related asymmetry in paraspinal muscle recruitment; however, the observed greater concave-side erector spinae RMS during lateral bending did not remain statistically significant after Holm correction and should therefore be interpreted as exploratory. Accordingly, although the coordinated thoracic rotation and associated muscle-activation patterns may potentially contribute to corrective rotational effects, neither corrective torque nor intervertebral stress redistribution was directly quantified for “Jiu Gui Ba Ma Dao.” These biomechanical interpretations should therefore be regarded as hypotheses derived from the kinematic and sEMG findings rather than as demonstrated mechanical effects.

“Lu Di” exhibits a more complex “spiral-lateral bending” 3D composite pattern, following a kinetic chain of “lumbar flexion leading to thoracic lateral bending.” This unique chain demands high multi-planar stability, as evidenced by the significant recruitment of the erector spinae and multifidus. During terminal lateral bending, the high activation of the convex-side muscles relative to the concave side creates an asymmetric torque differential. This synergistic contraction likely generates a transverse position-induced loading toward the concave side which, combined with rotation, forms a comprehensive 3D corrective moment (Kamal, 2024).

### 4.2 Clinical significance of stress equalization

The FEA results revealed a redistribution of intervertebral disc stress during the “Lu Di” movement. Specifically, the high stress concentration previously localized at the posterior concave annulus of the apical disc was reduced and redistributed toward the convex side, resulting in a more balanced stress distribution. This pattern is consistent with FEA findings from AIS bracing studies showing stress transfer from the concavity toward the convexity (Zhang et al., 2025a).

This redistribution may have two potential biomechanical implications.

First, reducing excessive stress concentration on the concave side may theoretically decrease repetitive mechanical loading associated with disc degeneration (Stokes and Iatridis, 2004; Adams et al., 1996).
Second, according to Wolff’s Law and stress-stimulus theories of tissue adaptation, repeated changes in mechanical loading may contribute to adaptive responses in spinal tissues (Wolff, 1986; Frost, 1983; Tam and Baar, 2025; Smit, 2024; Stokes et al., 2007). However, because the present FEA evaluated only the terminal static position of “Lu Di” and did not directly assess long-term tissue remodeling, these implications should be regarded as mechanistic hypotheses rather than demonstrated clinical effects.

### 4.3 Comparison with existing therapies and clinical application

Unlike previous biomechanical studies focusing primarily on passive external correction, such as bracing or surgery (Wang et al., 2014; Xu et al., 2025), or on relatively static corrective postures used in Schroth therapy (Zhang et al., 2025b; Day et al., 2019; Lehnert-Schroth, 1992; Park et al., 2018), the present study investigated active compound movements through an integrated kinematic, sEMG, and finite element framework. For “Lu Di,” FEA demonstrated redistribution of apical intervertebral disc stress at the terminal corrective position. In contrast, the biomechanical interpretation of “Jiu Gui Ba Ma Dao” was based on kinematic and sEMG findings, because finite element analysis was not performed for this maneuver.

The sEMG findings suggested different bilateral muscle-activation patterns between the two exercises. “Lu Di” showed higher convex-side multifidus RMS values, whereas “Jiu Gui Ba Ma Dao” showed a tendency toward greater concave-side erector spinae activation. However, these differences did not remain significant after Holm correction and should therefore be regarded as exploratory. Previous sEMG studies of physiotherapeutic scoliosis-specific exercises have likewise demonstrated exercise-dependent changes in paraspinal muscle activation, particularly during asymmetric corrective exercises (Wang et al., 2025).

Scoliosis-specific exercises such as the Schroth method have shown short-term improvements in Cobb angle, trunk rotation, and quality of life (Ceballos-Laita et al., 2023), and biomechanical evidence indicates that muscular forces may contribute to corrective spinal loading (Li W. et al., 2025). In addition, short-term scoliosis-specific exercise has been reported to improve spinal flexibility in patients with rigid AIS (Fan et al., 2025). In the present study, the distinct kinematic and sEMG patterns of the two exercises may therefore represent different neuromuscular and mechanical stimuli. For “Lu Di,” this interpretation is further supported by the modeled redistribution of apical disc stress, whereas the corrective torque and intervertebral stress effects of “Jiu Gui Ba Ma Dao” remain hypotheses because they were not directly evaluated by FEA.

Because only Lenke type 1 AIS patients were included, these findings do not support curve-type-specific exercise prescriptions and cannot be extrapolated to other curve patterns. Larger longitudinal studies are required to determine whether these preliminary biomechanical responses translate into structural or functional benefits.

## 5 Limitations and future directions

Several limitations should be acknowledged. First, the sample size was small, and the sEMG findings should be considered exploratory, particularly because several nominally significant differences did not remain significant after correction for multiple comparisons. Second, muscle forces were not explicitly simulated as active contractile elements in the finite element model; instead, muscle recruitment was inferred from sEMG amplitude and interpreted alongside the passive mechanical response. Third, finite element analysis was performed only for the terminal posture of “Lu Di,” and therefore captures neither dynamic loading throughout the movement nor the mechanical effects of “Jiu Gui Ba Ma Dao.” Fourth, kinematic inputs were derived from healthy adults and may not fully represent the segmental mobility and compensatory movement strategies characteristic of AIS patients. Fifth, the finite element model incorporated several structural simplifications, including the omission of gravitational loading, the absence of a compressive follower load, rigid sacral fixation, and the exclusion of a detailed rib cage. These simplifications may affect the predicted stress magnitudes and load-sharing patterns, and therefore the absolute stress values should be interpreted with caution. From a modeling perspective, global range-of-motion validation against published data supports the model’s overall flexibility and load transfer, but does not directly confirm the accuracy of internal stress predictions. Accordingly, disc and vertebral stress values are presented as relative comparisons across postural conditions, rather than as absolute physiological magnitudes. Future work should target stress-level validation using intradiscal pressure measurements, strain-based data, or imaging-derived patient-specific geometry, and should integrate synchronized subject-specific kinematics and sEMG into dynamic musculoskeletal–finite element frameworks in larger AIS cohorts.

## 6 Conclusion

This pilot study characterized the kinematic and neuromuscular responses of “Lu Di” and “Jiu Gui Ba Ma Dao” in patients with AIS. The two exercises exhibited distinct movement and muscle-activation patterns; however, concave-versus-convex sEMG differences were not significant after multiple-comparison correction and remain exploratory. FEA of “Lu Di” showed redistribution of apical disc stress, with reduced concave-side concentration and a more balanced distribution. For “Jiu Gui Ba Ma Dao,” kinematic and sEMG patterns suggest a potential biomechanical mechanism, but its disc stress effects were not directly evaluated. Integrating kinematic, sEMG, and (for “Lu Di”) FEA data provides a quantitative framework for studying TCM exercise biomechanics. These preliminary findings support further evaluation in larger cohorts with more comprehensive dynamic biomechanical models.

## Data Availability

The original contributions presented in the study are included in the article/Supplementary Material, further inquiries can be directed to the corresponding authors.
