# Supplementary material for: Biomechanical effects of traditional Chinese exercises on scoliosis: a combined finite element and surface electromyography analysis
Source: Front Bioeng Biotechnol. 2026 Oct 9;14:1895714. doi: 10.3389/fbioe.2026.1895714 (PMC13690814; doi:10.3389/fbioe.2026.1895714)
Supplement: Supplementary file 1 [file Supplementaryfile1.docx]

Supplementary Material

# 1 Supplementary Figures and Tables

## 1.1 Supplementary Figures

**Supplementary Figure 1.** Workflow of the L1–S1 spinal finite element model construction. (a–c) Segmentation of anatomical regions of interest (ROI); (d–e) Manual mask editing and refinement within Mimics software.

**Supplementary Figure 2.** Three-dimensional finite element model of the normal (intact) lumbar spine.

**Supplementary Figure 3.** Global deformation and stress contour maps of the scoliosis finite element model. (a) Initial state of the scoliosis model with the apical vertebra located at T7; (b–c) Finite element views of the spine at the terminal ranges of the prescribed exercises; (d–f) von Mises stress distributions (MPa) of the scoliosis model under flexion, left lateral bending, and left axial rotation, respectively.

**Supplementary Figure 4.**Equivalent (von Mises) stress contour maps of the intervertebral discs adjacent to the apical vertebra during different simulated movements.
